# Editorial: Peptide assemblies in nanotechnology

**DOI:** 10.3389/fmolb.2023.1281543

**Published:** 2023-08-30

**Authors:** Salvador Ventura, Marcelo J. Kogan, Rodrigo Diaz-Espinoza

**Affiliations:** ^1^ Institut de Biotecnologia I de Biomedicina (IBB) and Departament de Bioquímica I Biologia Molecular, Universitat Autònoma de Barcelona, Barcelona, Spain; ^2^ Departamento de Quimica Farmacologica y Toxicologica, Facultad de Ciencias Quimicas y Farmaceuticas, Universidad de Chile, Advanced Center for Chronic Diseases (ACCDiS), Santiago, Chile; ^3^ Departamento de Biología, Facultad de Química y Biología, Universidad de Santiago de Chile, Santiago, Chile

**Keywords:** peptide assembly, quorum sensing, functional amyloids, cicylic peptides, nanofibers

Nanotechnology is among the most promising and active research areas within the realm of molecular-based solutions for a wide diversity of challenges (Baig et al., 2021). Tailor-made supramolecular assemblies can lead to novel materials at the nanoscale level with unique emergent properties such as catalytic reactivity and high chemical and mechanical stability (Mitchell et al., 2021). Nanostructures assembled with components derived from or based on biological molecules have emerged as a prominent alternative to chemical building blocks, since they can act as biocompatible nanomaterials that can safely operate in biological environments as well as exhibit functionalities that can mimic and sometimes match those of biomolecules (Stater et al., 2021). Among these, peptides are highly valuable building blocks since they can be easily synthesized in both laboratory settings and on a larger scale. Moreover, they can assemble into supramolecular structures that can exhibit some of the functions observed with their macromolecular counterparts: proteins (Yu et al., 2016; Levin et al., 2020). Peptides can also be chemically engineered through reliable and relatively easy means, allowing the emergence of novel functions or properties in their assembled state, which can dramatically increase their application (Luo et al., 2021). In addition to rational design approaches, studying naturally occurring peptide assemblies can provide insights into their physiological functions and their potential as future nanomaterials. This can in turn lead to uncover novel therapeutic targets or pathological mechanisms.

In this Research Topic, we have published four original articles on the field of peptide assemblies and their potential applicability as novel bionanomaterials. The Research Topic includes a wide selection of approaches, from rational design of peptides to achieve novel functions in the assembled state to naturally occurring supramolecular assemblies involved in both antimicrobial activity and pathogenic infection. For instance, as part of the immune response certain cell types can produce peptides involved in antimicrobial defense that can exhibit a wide diversity of antimicrobial mechanisms. Interestingly, intermolecular assemblies of these peptides are increasingly being recognized as relevant players in some of those mechanisms. In the first article by Zsila et al., the interactions between antimicrobial peptides (AP) and small molecules involved in microbial quorum sensing (QS) are explored. QS is a particularly relevant inter-microbial communication system that can regulate an array of microbial functions, including biofilm formation and stabilization, which is a key element in pathogenic bacterial infection. In this work, the known human cathelicidin AP (LL-37) is shown to engage in specific interactions with a quinolone signal molecule from *Pseudomonas aeruginosa*, which is an essential component of QS during pathogenic infection. The interactions induce the rapid assembly of LL-37 into supramolecular arrays to which many quinolone signals are bound in a regular fashion. The resulting aggregates are only formed in presence of the quinolone signal and therefore appears as specific to this molecule. Since peptide assemblies are typically considered much more stable forms than their monomeric counterparts, it is then tempting to speculate that this specific assembly may be a natural element of the antibacterial activity of the LL-37, possibly causing an irreversible trapping of the quinolone signal and thus hampering the pathogenic response. This study could therefore open novel avenues to explore the role of peptide self-assembly in immune mechanisms.

Experimental approaches based on peptide libraries such as yeast-based phage display (YPD) can be excellent tools to screen for bioactive peptides with diverse applications. In the work by Rosa et al., a novel cyclic peptide is designed using a modified YPD approach. The cyclization of peptides brings important advantages such as increased biostability to proteases and decreased flexibility. Using the positive regulator of galactose metabolism GAL4 as a bait protein, the authors identified a peptide that induced a GAL4-specific decreased accumulation of galactose-related cytotoxic metabolites. The use of these libraries may also serve in the future to screen for peptides that through assembly might lead to novel nanomaterials.

Rayan and co-workers investigated the diverse thermostability of functional amyloids. They could associate the atomic structure of a fibril to its thermostability, as illustrated for PSMα1 and PSMα3, two *staphylococci* peptides with similar sequences but distinct fibril architectures. The study indicates that the heat responses of these peptides are determined by their specific internal bonds. PSMα3’s configuration potentially enables it to engage with cell membranes, resulting in its detrimental cellular impact. In contrast, PSMα1 establishes sturdier structures, bolstering bacterial biofilm stability. Notably, the research shows that environmental factors can influence the structure and thermostability of the PSMα1 and PSMα3 fibrils. This understanding offers a foundation for crafting innovative active peptides responsive to specific external cues, which could have implications for nanotechnology applications.

In the article published by Liang and co-workers the authors developed a peptide nanofiber self-assembled from spiropyran (SP)-modified β-sheet-forming peptides, which can be reversibly polymerized/depolymerized by light due to a photoisomerization process. The modified peptide was encapsulated within spherical giant vesicles made of phospholipids, serving as artificial cell models. By application of light it is possible to manipulate the morphology due to the photoisomerization process that leads to a dissociation of the peptide. These dynamic morphological changes could be used as a potential tool to remotely control cellular functions.

The articles presented in the Research Topic reinforce the idea that designed and naturally occurring peptide assemblies can serve as a rich source to inspire the development of novel nanomaterials. Beyond the many practical advantages of using peptides, the assembled state can provide stability and emergent functionality, two features especially coveted for applications in nanotechnology.
